# Seasonal Variability of Human Enteric Viruses Discovered in Food Production Mussels (*Mytilus galloprovincialis*) Farmed in the Central Adriatic Sea (Italy)

**DOI:** 10.3390/foods13203329

**Published:** 2024-10-20

**Authors:** Gianluigi Ferri, Vincenzo Olivieri, Alberto Olivastri, Chiara Di Vittori, Alberto Vergara

**Affiliations:** 1 Department of Veterinary Medicine, Post-Graduate Specialization School in Food Inspection “G. Tiecco”, University of Teramo, Strada Provinciale 18, 64100 Teramo, Italy; violivie1956@libero.it (V.O.); cdivittori@unite.it (C.D.V.); avergara@unite.it (A.V.); 2 Hygiene of Food of Animal Origin (SIAOA) Veterinary Public Service, AST Ascoli Piceno, 63100 Ascoli Piceno, Italy; albertoolivastri@libero.it

**Keywords:** RNA viruses, foodborne pathogens, *Mytilus galloprovincialis*, molecular biology, water temperature, seasons

## Abstract

Among the different naked and quasi-enveloped viruses, the hepatitis A virus (HAV), hepatitis E virus (HEV), and norovirus genogroups I and II (NoV GI and NoV GII) are considered the main microbiological noxae involved in foodborne outbreaks. Mussels can harbor pathogens in their tissues. In addition to epidemiological attention, marine water temperature changes are considered a crucial variable influencing viral loads. This study aimed to biomolecularly screen 1775 farmed mussels (*Mytilus galloprovincialis*) for viral ribonucleic acid (RNA) sequence detection (belonging to the HAV, HEV, and NoV GI and GII genogroups) in three different sampling periods (spring, summer, and winter), with the mussels collected from three farms located in the Central Adriatic Sea (Italy). The results showed that 10.42% of the screened animals harbored at least one type of pathogen RNA, more specifically, 5.35% HEV, 4.51% NoV GI, and 0.56% HAV. The highest genetic equivalent (GE) amounts were majorly observed in the winter season (NoV GI 1.0 × 10^3^ GE/g and HEV 1.0 × 10^2^ GE/g), resulting in statistical differences when compared to summer and spring (*p*-value: <0.001). The original data obtained serve to bring scientific attention to the possible influence of environmental and climatic aspects on viral loads, highlighting the crucial role played by biomolecular assays as preventive medicine tools.

## 1. Introduction

Naked or quasi-enveloped viruses [1] such as the hepatitis A virus (HAV), hepatitis E virus (HEV), and noroviruses GI (NoV GI) and GII (NoV GII) consistently gain scientific attention due to their wide environmental diffusion. They represent crucial public health concerns because infections can occur through the ingestion of raw or under-cooked contaminated food matrices of animal origin. Bivalve lamellibranches have been considered as target animal species for the rooted persistence of viral pathogens [2].

The *Mytilus* genus has been studied in many scientific investigations for its harboring and bioaccumulation of different microbiological noxae (viruses, bacteria, etc.) [3]. The US Food and Drug Administration data confirm the European Food Safety Authority’s observations about the growing infection cases mainly caused by viral foodborne pathogens such as HAV, HEV, and NoV GI and GII [4]. The absence of established legal quantitative limits to viral genome detection (with special regard to the European Reg. No. 2073/2005) in different animal-origin food matrices has increased the scientific necessity of providing evidence and trying to fill the above-mentioned gap for the legislator [5].

In the European scenario, including the Italian one, the HAV, HEV, and NoV RNA sequences have been largely amplified in bivalve species, with special regard to *Mytilus galloprovincialis*. These viruses, harbored in mussels’ tissues, have mainly been discovered in the southern Italian regions. NoV and HAV RNA fragments were observed in Campania (NoV GI: 10.8%, NoV GII: 39.7%, and HAV: 8.9%) by Fusco et al. [6] and in Sicily (NoV GI: 2.9%, NoV GII21.7%) by Purpari et al. [7], as well as in the Tyrrhenian Sea [8]. In the Adriatic Sea, La Bella et al. [9] observed NoV GI RNA at levels starting from 1.6% in *Mytilus galloprovincialis* in the Apulia region, and they were found at a level of 1.7% by Savini et al. [10] in the Molise region. On the other hand, HEV RNA was found to be marginally amplified (prevalence value: 0.89%) in mussels (farmed in the Apulia region) along the coasts belonging to the Adriatic Sea (Italy) by La Bella et al. [11].

The epidemiological interest in viral circulation has been coupled with another important variable, represented by the climatic factor, which is considered the determining aspect with regard to the amounts of viral loads in the marine environment [12]. In Europe, Errani et al. [13] discovered significantly higher NoV and HAV RNA quantities in mussels (36.7%) during the winter season than during the spring and summer seasons. They suggested that cold temperatures play crucial roles in naked viruses’ survival in marine environments, highlighting the importance of bivalves as target animal species.

Based on the above, the present scientific investigation aimed to biomolecularly screen mussels (*Mytilus galloprovincialis*) for the viral RNA detection of the following naked or quasi-enveloped viruses: HAV, HEV, and NoV GI and GII. It focused on a crucial variable represented by seasonal changes in the marine water temperature. More specifically, one-step real-time polymerase chain reaction (RT-qPCR) and nested reverse-transcription polymerase chain reaction (nested RT-PCR) assays were used to verify a scientific hypothesis about the influence of marine water temperatures (related to the seasonal typical modifications of the Mediterranean European countries) and the respective viral loads in mussels’ tissues. For this purpose, a total of 1775 *Mytilus galloprovincialis* samples were collected from three different mariculture farms (located in the Central Adriatic Sea along the coasts of three Italian regions: Marche, Abruzzo, and Molise) during three different collection moments: spring (March 2023), summer (June 2023), and winter (January 2024).

Based on the obtained evidence, this study aimed to provide original data about a less-investigated marine area (the Central Adriatic Sea), trying to demonstrate the *domino effects* of climate variables on marine environments and, consequently, on microbiological viral populations in animals used for food production.

## 2. Materials and Methods

### 2.1. Sample Collection

**Figure 1:**
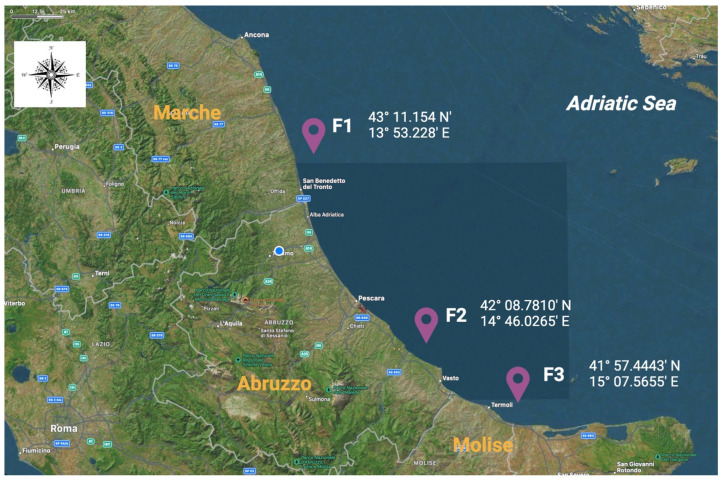
Geographical distributions and their respective georeferentiations of the screened mariculture farms involved in the present biomolecular investigation. **F1:** farm 1, located in the Marche region; **F2:** farm 2, located in the Abruzzo region; **F3:** farm 3, located in the Molise region.

Sampling activities lasted from March 2023 to February 2024, and a total of 1775 bivalve mussels belonging to the *Mytilus galloprovincialis* species were included in the scientific investigation. The screened animals were farmed in three different mariculture sites, which were located along the coasts of Marche (named as F1), Abruzzo (F2), and Molise (F3) regions, which are characterized by low industrialization and anthropization levels in the Adriatic Sea, as illustrated in Figure 1.

In all three mariculture sites (F1, F2, and F3), mussels were farmed following the longline system; water microbiological quality was classified as *A-category*, which means that *Escherichia coli* amounts were at less than 230 strains/100 g of tissues and liquid, as reported in the European Regulations (EU Reg.) No. 853/2004 and N. 627/2019. All animals conferred for the virological screenings bypassed the so-called purification phase and were ready for commerce. These subjects gained average final seizures of 7.2 ± 0.4 for length and 2.9 ± 0.3 for width; they were collected in an alive state, and their transportation was performed under refrigerated conditions, as defined by the EU Reg. No. 853/2004. After receipt, the samples were directly conferred for laboratory processing.

Specimen collections were designed and performed during three different seasonal moments, according to the southern European climate peculiarities. More specifically, between spring, summer, and winter, there are consistent temperature variabilities ranging from 6 to 12 °C [14]. Based on these climatic characteristics, collection months were established, and each farm was sampled three times (S1, S2, and S3). For each sampling step, the registered data included the number of collected subjects, georeferentiation, and water temperatures. A detailed representation is illustrated in Table 1.

The foodborne pathogens studied in the present study were HAV, HEV, and NoV GI and GII. In more detail, the RNA amplification of specific genomic regions was the main target of the biomolecular assays, as described in the proper subheading.

The first analytical step (at the laboratory level) was represented by tissue collection with special regard to the mussels’ gastroenteric glands. They were sterilely collected using mono-usage scalpels (Monopec Scalpels, Thermo Fisher Scientific^TM^, Waltham, MA, USA) and introduced to sterile vials (Nunc^TM^, 50 mL Conical Sterile polypropylene Centrifugate Tubes; ThermoFisher Scientific^TM^, Waltham, MA, USA) with a final volume of 50 mL, as described by the International Standard ISO 15216:2018 [15] (used for viral foodborne pathogen detections). Each vial contained five subject/farm/collection steps (S1, S2, or S3), and based on this, a total of 355 pools were obtained from 1775 mussels, as described in detail in Table 1. More specifically, 151 came from F1, 91 from F2, and 113 from F3. The second step involved a tissue homogenization procedure, which was performed by using the T18 digital Ultra-Turrax^®^ (Staufen, Germany). Each pool was homogenized for 3 mins. The third one was refrigerated centrifugation using the following parameters: 4000 g for 20 min. After centrifugation, the obtained supernatant was collected and filtered with final aliquots of 5 mL/pool, as previously described by Szabo et al. [16]. Finally, all specimens were stored at −80 °C till biomolecular assays were performed.

### 2.2. RNA Extraction and Biomolecular Assays

RNA extraction was performed following the TRIzol LS method (Invitrogen, Ltd., Paisley, UK). In more detail, from each pool (5 mL/pool), a volume of 500 µL (v1) was collected. In agreement with the adopted protocol, 250 µL of TRIzol was added to v1 and centrifugated at 12,000× *g* for 15 min; the obtained supernatants were removed, and pellets were successively washed by consecutively adding 500 µL of two alcoholic solutions: isopropyl (75%) and ethylic (75%) alcohols. A last centrifugation at 7500× *g* for 5 mins was included, permitting us to obtain final pellets resuspended in 50 µL of RNase-free water (Invitrogen UltraPure DNase/RNase-Free Distilled Water, ThermoFisher^TM^, Waltham, MA, USA). All extracts were finally stored at −80 °C until the biomolecular analyses were performed.

Biomolecular screenings included both qualitative and quantitative assays, which were performed as nested RT-PCRs and one-step real-time RT-qPCR. Qualitative versions were realized in final reaction volumes of 25 µL using specific kits Qiagen^®^ OneStep RT-qPCR Kit (Hilden, Germany) for reverse transcription (first reaction) and Green Master Mix Promega^®^ (Madison, WI, USA) for the second one. The primers and thermocycler settings matched the referenced scientific papers, as illustrated in Table 2.

Qualitative nested RT-PCR products were successively loaded onto agarose gels (1.5% and 2.0% as concentrations), and the expected amplicons, located at the specific length, were compared to different DNA ladders (50 bp and 100 bp) (Genetics, FastGene^®^, Düren, Germany). The suspected positive bands (that represented pools) were sequenced using the Sanger method realized in collaboration with BioFab Research (Rome, Italy). The nucleotide similarity evaluations were performed by loading the obtained sequences onto the BLAST system platform (https://blast.ncbi.nlm.nih.gov/Blast.cgi?PROGRAM=blastn&BLAST_SPEC=GeoBlast&PAGE_TYPE=BlastSearch) (accessed on 3 March 2024). Regarding HEV, the suspected sequences were also loaded for similarity tests using the HEV-Typing tool (https://www.rivm.nl/mpf/typingtool/hev/) (accessed on 15 February 2024).

The quantitative biomolecular assays aimed to detect genetic regions belonging to the genomes of the studied viruses (HAV, HEV, and NoV GI and GII). These analyses were performed using one-step real-time RT-qPCR using the GENE UP^®^ System (bioMérieux, Paris, France). All final reaction volumes for all three considered pathogens were 25 µL, formed of 20 µL of biomolecular reagents and 5 µL of extracted RNA, in agreement with the manufacturer’s instructions for the commercial kits (ceeramTools—Thermo Fisher Scientific^TM^, Waltham, MA, USA). The nucleotide quantity determinations were obtained via the realization of standard curves at known concentrations starting from an initial amount of 1.0 × 10^6^ to 1.0 × 10^1^ genome equivalent/g of tissue (GE/g) performing 10-fold dilutions. These versions were part of the used kits ceeramTools—Thermo Fisher Scientific^TM^ (Waltham, MA, USA). The established limit of quantification (LOQ) was 1.0 × 10^0^ GE/g for HAV, HEV, and NoV GI and GII. The thermocycler setting was the same for all four studied viral pathogens. It started with initial RNA reverse transcription at 45 °C for 10 min, followed by 95 °C for 10 min, 40 cycles characterized by 95 °C for 15 s and 60 °C for 45 s. Positive controls were included for all screenings and were included in the commercial kits ceeramTools Thermo Fisher Scientific^TM^ (Waltham, MA, USA). Sterile RNase and DNase-free water (Invitrogen UltraPure DNase/RNase-Free Distilled Water, ThermoFisher^TM^, Waltham, MA, USA) was used as the negative version.

### 2.3. Statistical Analysis

All statistical tests were performed using IBM^®^ SPSS 20.0 Software (SPSS, Chicago, IL, USA). The two-tailed paired *t*-test was selected as a reference statistical test for two different considerations. The first was to compare the amounts of the discovered amplicons (expressed as GE/gram of tissue) per each collection area (F1, F2, and F3). The second compared the viral genetic amounts (belonging to HAV, HEV, NoV GI, and NoV GII) to the three different collection moments: S1, S2, and S3. In more detail, the considered dependent variables were the four screened viruses (HAV, HEV, and NoV GI and GII). The results were considered significant if the *p*-values were <0.05. The confidential interval at 95% (CI: 95%) was calculated, when applicable, for all percentages.

## 3. Results

In the collection period (2023/2024), 1775 bivalve lamellibranches (*Mytilus galloprovincialis*) or 355 pools were collected in three different farms during the spring, summer, and winter seasons from mariculture realities located along the coasts of Marche (F1), Abruzzo (F2), and Molise (F3) regions in the Adriatic Sea. From a general perspective, 37 out of 355 or 10.42% (CI 95%: 7.24–13.60%) of the screened pools harbored at least one of the screened pathogens (RNA-specific fragments, as previously indicated in the Section 2). Indeed, ORF-1 and ORF-2 (concerning HEV), VP1/2A junction (HAV), and Capsid N/S domain (NoV GI and GII) were generally amplified in the respective positive pools.

Among the three detected viral pathogens, HEV and NoV GI were mostly discovered; indeed, 19 out of 37 or 51.35% (CI 95%: 35.25–67.45%) of the screened positive pools harbored HEV RNA, and 16 out of 37 or 43.24% (CI 95%: 27.28–59.20%) were positive for NoV GI RNA detection. On the other hand, HAV was discovered in 2 out of 37 or 5.40% (CI 95%: 0.30–10.50%) of the positive subjects. None of the screened samples harbored parts of NoV GII RNA.

The codetection of viral RNA was observed in 2 out of 37 or 5.40% (CI 95%: 0.30–10.50%) of the positive pools (coming from F2) harboring HEV and NoV GI RNA parts, and parts of the HAV and NoV GI genomes in another one pool belonging to animals were collected in F3.

Based on the geographical variable, the discovered positive pools were mainly observed in F3, presenting 15 out of 37 (40.54%; CI 95%: 24.72–56.36%) of the amplified viral RNA fragments; a total of 13 out of 37 or 35.13% (CI 95%: 19.75–50.51%) and 9 out of 37 or 24.32% (CI 95%: 10.50–38.14%) were discovered from F1 and F2, respectively. From a general perspective, the comparisons between the different viral RNA amounts belonging to the three considered viruses (expressed as GE/g of sampled tissue) and the farm localizations presented significant statistical differences when comparing F3–F1 (*p*-value 0.02) and F3–F2 (*p*-value: 0.001). In more detail, HEV RNA was mostly amplified in pools coming from F2 with the following value: 10 out of 19 (52.63%; CI 95%: 30.18–75.08%). Regarding HAV, parts of its RNA were only amplified from mussels farmed in F3, and NoV GI was mainly discovered from F3 specimens: 10 out of 16 or 62.50% (CI 95%: 38.78–86.22%). A detailed schematic representation of the viral pathogen distribution is illustrated in Table 3.

**Figure 2:**
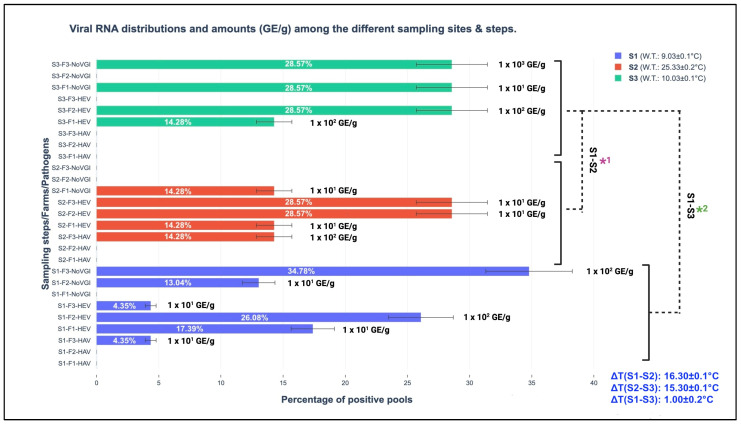
Viral RNA distributions and amounts (GE/g) among the different sampling sites and steps. **S1:** sampling 1; **S2:** sampling 2; **S3:** sampling 3; **GE/g:** genome equivalent per gram of screened tissue; **F1:** farm 1 (Marche region); **F2:** farm 2 (Abruzzo region); **F3:** farm 3 (Molise region); **W.T.:** water temperature (°C); **ΔT:** thermal water differences among the seasons; S1-S2*1 and S1-S3*2: *t*-test comparing the total obtained viral amounts (GE/g) for the sampling steps.

Considering marine water seasonal temperature changes in the sampled sites (as schematically reported in Table 1), the screened RNA belonging to the studied viral pathogens was different. Among the three different seasonal sampling steps, the spring version was mostly represented by the wide detection of parts of the three viral genomes; indeed, 23 out of 37 or 62.16% (CI 95%: 46.54–77.78%) were amplified during S1, and 7 out of 37 or 18.92% (CI 95%: 6.30–31.54%) were amplified during S2 and S3. The performed *t*-test discovered statistically significant differences comparing the obtained total viral amounts (HAV, HEV, and NoV GI, expressed GE/g of screened tissue) among the respective sampling steps (S1, S2, and S3) presenting *p*-values of <0.001; in more detail, they were observed between the following sampling versions: S1–S2 and S1–S3. A detailed representation of viral pathogen distributions among the different sampling steps and their respective GE/g of screened tissue are graphically illustrated in Figure 2.

Regarding HEV sequences, the BLASTN and HEV NET typing tools showed high nucleotide similarities (nt. ID: 99.0%) with genotype 3 subtype c. Sequencing and the respective alignment evaluations permitted the discovery of high similarities (nt. ID: 98.0%) to HAV I and NoV GI regarding suspected positive pools.

## 4. Discussion

The present biomolecular investigation involved the most frequently discovered enteric viral pathogens, such as HAV, HEV, and NoV GI and GII, amplified from bivalve lamellibranches, including the *Mytilus* genus. The screened animals were farmed at three mariculture sites (F1, F2, and F3) along the coasts of the Marche, Abruzzo, and Molise regions (Central Adriatic Sea). The performed one-step real-time RT-qPCRs and nested RT-PCR assays aimed to focus scientific attention on the viral genetic fragments’ diffusion and their relative distributions in the studied marine environments. This above-mentioned reasoning also involved another crucial variable represented by the marine water temperature changes associated with seasons. Another crucial factor that must be considered for viral diffusion (HAV, HEV, and NoV GI and GII) is represented by wastewater management. In more detail, it refers to the efficacy of these plants in purifying waste coming from hospitals and zootechnic sectors; indeed, the filters used (in developed countries) present difficulties in significantly reducing viral loads [1].

These aspects are linked to sanitary repercussions, and from a legal perspective, they need to be considered due to the current absence of legal parameters on viral pathogens in food matrices. Indeed, it is confirmed by the lack of scientific limits in the current European Reg. No. 2073/2005 except for NoV GI and GII in mixed barriers (both for Food Safety Criteria and Hygiene Processes Criteria) [23]. The scientific literature and the relative evidence demonstrated sanitary implications if any specific animal-origin food matrices were consumed raw. Therefore, the introduction of further criteria will represent the proper legal behavior to preserve consumer and environmental health [24,25]. Following these bimodal reasonings, mussels were indicated as target animal species because they are considered environmental biosentinels (expressions of the so-called bioaccumulation phenomenon) [2] with special regard to viral pathogens [12].

From a European perspective, NoV GI and GII and HAV have been widely discovered from different bivalve lamellibranches (including mussels) when compared to HEV virions that have low detection rates and few investigations [26,27].

In the present study, 10.42% (CI 95%: 7.24–13.60%) of the 355 screened pools (which represented 1775 animals) harbored RNA fragments belonging to at least one of the investigated viral pathogens, as previously described in the Section 3. The obtained percentage value (10.42%) was in line with the general detection percentage of 10.64% reported by Fusco et al. [28] from mussels (*Mitilus galloprovincialis*) farmed along the coasts of the Tyrrhenian Sea (Campania region). In more detail, HEV RNA was not amplified by Fusco et al. [28]; however, they conversely discovered other viral genetic determinants belonging to other pathogens (such as aichivirus, astrovirus, rotavirus, and sapovirus) that were not included in the present investigation. The comparisons with other studies demonstrated that the present percentage was higher than the 2.25% of positive specimens (to at least one pathogen) described by Pavoni et al. [25]. They also observed multiple NoV/HAV contaminations from 22.59% of the positive samples. In the present investigation, a similar coexistence of NoV/HAV was also observed in one pool coming from F3. Furthermore, another discovered coupling was NoV/HEV in one case from animals farmed in F2 (see the Section 3).

The differences among the prevalence values find their scientific rationale based on the different anthropic pollution of the screened areas, as suggested by Upfold et al. [29]. Although all screened animals (tested in the previously mentioned studies) were collected from mariculture farms (the waters of which were classified as A-category in agreement with the European Reg. No. 853/2004), the prolonged viral persistence in mussels’ tissues due to the consistent environmental impact of wastewater has been confirmed. Indeed, due to their structural characteristics, naked or quasi-enveloped viral RNA pathogens gain intracellular protection from marine ecosystems [30].

Among the studied viral pathogens, HEV RNA was mainly amplified (19 out of 37 or 51.35%; CI 95%: 35.25–67.45%) from the specimens when compared to NoV GI RNA (16 out of 37 or 43.24%; CI 95%: 27.28–59.20%) and HAV (2 out of 37 or 5.40%; CI 95%: 0.30–10.50%). None of the pools harbored NoV GII, as described in the Section 3. In the marine Italian scenario, the discovered prevalence values/viral pathogens resulted in differences when compared with other similar investigations. In more detail, 19 out of 355 or 5.35% (CI 95%: 3.01–7.69%) of the positive pools harbored HEV RNA fragments (ORF-1 and ORF-2); this value was higher than those discovered from mussels in the Sicily region, where 2.6% was found by La Rosa et al. [31], 0.9% was found by Purpari et al. [7], and Macaluso et al. found none in the samples [8].

The Adriatic Sea, conversely, has seen few investigations, and the first HEV RNA detection, observed in 0.89% of screened bivalves (*Mytilus galloprovincialis*, *Crassostrea gigas*, etc.) was reported by La Bella et al. [11] in the southeast part of the Adriatic Sea along the Apulian coasts. These differences could find scientific explanation due to the peculiar marine currents that characterize the studied marine areas (Central Adriatic Sea), which can facilitate viral bioaccumulation due to these favorable environmental conditions [13]. Another crucial variable is represented by marine water microbiological quality, which has consistent sanitary implications. Indeed, the above-mentioned studies performed in the Sicily [7,31] and Apulia [11] regions included bivalves farmed in mariculture areas classified in the B category (as described in the EU Reg. No. 853/2004), which is in contrast to the *A category* waters in the present study. These results, both in the present study and in other studies, confirm the different natural behavior of naked viruses harbored (several-fold) in bivalve tissues when compared to the well-known reference bacterial parameter Escherichia coli [32] (which has inspired the reference values reported in European Reg. No. 853/2004).

Regarding HEV, the discovered and involved genotype, based on the results obtained from the phylogenetic analysis, was 3c, as described in the Section 3. The one-step real-time RT-qPCR assays showed different distributions of viral RNA amounts among the three different sampling moments (S1 (spring), S2 (summer), and S3 (winter)), as schematically illustrated in Figure 2 (Section 3). In more detail, in the winter season, HEV RNA fragments were quantified at 1.0 × 10^2^ GE/g both in F1 and F2, resulting in higher values than 10^1^ GE/g (spring collection) in F1 and F3, 1.0 × 10^2^ GE/g in F2, and summer 1.0 × 10^1^ GE/g (F1, F2, and F3). Between S3 and S2, a statistical difference was observed (*p*-value < 0.001). Between S1 and S3 at the same farm level (F2, located in the Abruzzo region), HEV RNA amounts of 1.0 × 10^2^ GE/g were maintained, as illustrated in Figure 1. This last-mentioned result finds its first scientific fundamental from HEV structural peculiarities, which have been demonstrated to be resistant against different extreme environmental conditions (i.e., strong acid solutions, per-osmolar conditions, etc.) [33]. Secondly, no consistent water temperature changes between S1 and S3 (S1: 9.03 ± 0.1 °C and S3: 10.03 ± 0.1 °C) were observed in the present study. Indeed, the obtained ΔT was ∆T (S1–S3): 1.00 ± 0.2 °C, presenting a lower expected value in the seasonal transitions (between 6.0 to 12.0 °C), as described by Flannery et al. [14] in order to have significant influences on viral loads. Cold water temperature maintenance permitted high GE/g harboring in mussels from S1 to S3. This condition was also described by Errani et al. [13], who identified high viral genome contents (belonging to human enteric viruses such as HAV and NoV) during the winter season from mussels (*Mytilus* genus) farmed along the coasts of Ravenna (Emilia-Romagna region, Italy). This concept highlights the crucial role of water temperature in viral survival and determining environmental resistance and persistence [34].

Regarding NoV, the discovered genogroup was represented exclusively by NoV GI, which was qualitatively amplified from 16 out of 37 or 43.24% (CI 95%: 27.28–59.20%) from the positive pools (with a general prevalence of 16 out of 355 or 4.51% (CI 95%: 2.35–6.67%)) in the three different sampling moments (S1, S2, and S3). Sequences belonging to NoV GI were mainly amplified during S1, at 11 out of 16 or 68.75% (CI 95%: 46.04–91.46%), and during S3, at 4 out of 16 or 25.00% (CI 95%: 3.78–46.22%), but not during summer (S2). The calculated *t*-test demonstrated statistically significant differences between the S1 and S3 (*p*-value: <0.02), S2 and S3 (*p*-value: <0.001), and S1 and S2 (*p*-value: <0.001) NoV GI RNA amounts, respectively.

When comparing these results with the scientific literature, it emerged that the obtained percentage (calculated among the total of 355 pools) of 4.51% for NoV GI (alignments showed high nt. ID of 99.0%) was higher than the values of 1.6% belonging to the same genogroup (I) discovered in the southern Adriatic Sea along the Apulian coasts by La Bella et al. [9] and 1.7% observed by Savini et al. [10] in the Molise region. It is important to mention that NoV GII was never amplified in the present investigation, which is in line with the genetic absences of NoV GI and GII from mussel samples observed by Purpari et al. [7] in the Sicily region. In contrast to these reported data, La Bella et al. [9] discovered genogroup II in 12.22% of screened animals. High NoV prevalences were also described in other Italian marine areas, e.g., the northern parts of the Adriatic and Ligurian Seas, characterized by 40.00% positive bivalves, more specifically, 13.0% for NoV GI and 46.0% for NoV GII [35]. Finally, 18.00% of specimens belonging to the *Mytilus* genus were positive for NoV GI RNA in the Tyrrhenian Sea [2].

From a quantitative perspective, NoV GI saw higher amounts in S3 (winter season), with a value of 1.0 × 10^3^ GE/g, and an absence of RNA in S2 (summer); 10^2^ GE/g was observed in S1 in the same farm (F3). A schematic illustration of the GE/g distributions among the farm sites, pathogens, and seasons is provided in Figure 2. The observed seasonal NoV behavior confirms the scientific hypothesis about the importance of water temperature variability for viral loads. Indeed, consistent water thermal changes such as ∆T (S1–S2): 16.30 ± 0.1 °C and ∆T (S2–S3): 15.30 ± 0.1 °C were observed in two specific moments between the warm and cold seasons. The results obtained from the one-step RT-qPCR assays support and confirm that low temperatures (between January and March in Mediterranean Europe) provide favorable environmental conditions for NoV survival and environmental persistence, with this also conserving its infectivity with special regard to naked viruses or quasi-enveloped versions, as suggested by Choi and Kingsley [36].

Among the three screened pathogens, HAV RNA was amplified in 2 out of 37 or 5.40% (CI 95%: 0.02–10.78%) of the positive pools collected during S1 and S2 in the same farm (F3) (as schematically illustrated in Figure 2). None of the pools sampled in S3 were positive (see the Section 3). The obtained prevalence of 5.40% was lower when compared with other studies. Indeed, along the coasts of the Campania region in the Tyrrhenian Sea, various research groups discovered the following HAV RNA prevalence values: 8.90% described by Fusco et al. [6], 13.67% by La Rosa et al. [31], and 22.70% bivalves (including different species such as *Mytilus galloprovincialis*, *Solen vagina*, *Venus gallina*, and *Donax trunculus*) [37].

Unlike the Tyrrhenian Sea, the Adriatic Sea has only been marginally investigated for HAV RNA; indeed, in its southern area, the HAV genome was not amplified from bivalves, as reported by La Bella et al. [9], who screened many species (e.g., *Mytilus galloprovincialis*, *Venus gallina*, and *Ostrea* spp.).

From a seasonal perspective, the amplified HAV RNA regions presented higher amounts (1.0 × 10^2^ GE/g) in the summer season (S2) than in the spring season (S1 1.0 × 10^1^ GE/g), with an absence in winter (S3) (see Figure 2). This observed trend is in contrast to the environmental behavior described by Errani et al. [13], who identified cold months as target periods for viral persistence. These differences find their fundamental basis in anthropic pollution associated with the peculiarity of marine currents that characterize F3. As a result, the consistent demographic incomings for touristic purposes along the coasts of the screened Adriatic Sea parts contribute to increasing possible viral circulation in the marine environments. However, high water temperatures (summer season) reduce their survival, which is conversely improved by cold temperatures, and the consequential bioaccumulation and persistent exposure to virions cover crucial roles representing risks for consumers [38]. The strict linkage between climate parameters and marine environments has important repercussions on viral life cycles, with special regard to naked or quasi-enveloped viruses. Based on the obtained evidence, this investigation also helps to emphasize and enforce (in line with other similar studies) the strategic role of biomolecular surveillance as a preventive tool to guarantee human, animal, and environmental health. The new frontier to prevent/reduce viral diffusion (obtained from human and zootechnic sources) in the marine environment is represented by wastewater plants. Indeed, at these levels, innovative filters with nanopores will have consistent impacts on viral load reduction. The most probable solution will be provided by the scientific combination of animal medicine and environmental engineering to guarantee safe foodstuffs for consumers [39].

## 5. Conclusions

The studied seasonal behavior of the main enteric naked or quasi-enveloped viruses (HAV, HEV, and NoV GI and GII) permitted us to obtain original data on viral RNA sequence circulation, harbored by farmed *Mytilus galloprovincialis* subjects, at three different mariculture sites (F1, F2, and F3) located along the coasts of the Central Adriatic Sea. The discovered influence of seasonal variation, and, more specifically, water temperature change, permitted the discovery of statistically significant differences in pathogen genomic amounts and distributions (with special regard to HEV and NoV GI) by comparing winter (S3), spring (S1), and summer (S2) values. Furthermore, this last aspect is directly linked to marine current peculiarities, which can be fundamental for the bioaccumulation and sedimentation of many particles (including viruses) in many seafood species, with great attention focused on bivalve lamellibranches, as suggested by Yang et al. [11]. The observed geographical differences are also influenced by the anthropic pollution levels in specific urban regions, and this has been demonstrated to be directly dependent on wastewater management, which is responsible for viral spread in the environment.

Finally, it is important to mention the necessity of specific legal parameters, such as quantitative cut-offs, to provide safe seafood matrices for final consumers and also defend marine environmental health.

## Figures and Tables

**Table 1:** Sample subjects from 2023–2024 included in the present scientific investigation.

Sampling Steps	No. of Sampled Subjects	Georeferentiation	Italian Region	Temperature
**S1** (March 2023)	110	Casalbordino (CH): 42° 08.7810’ N; 14° 46.0265’ E	Abruzzo	9.5 ± 0.2 °C
**S2** (June 2023)	110			24.2 ± 0.1 °C
**S3** (January 2024)	235			10.1 ± 0.1 °C
**Sampled animals: 455**				
**S1** (April 2023)	160	Termoli (CB): 41° 57.4443’ N; 15° 07.5655’ E	Molise	8.9 ± 0.1 °C
**S2** (June 2023)	155			25.1 ± 0.2 °C
**S3** (January 2024)	250			11.3 ± 0.1 °C
**Sampled animals: 565**				
**S1** (March 2023)	255	Torre di Palme (FM): 43° 11.154’ N; 13° 53.228’ E	Marche	8.7 ± 0.1 °C
**S2** (July 2023)	250			26.7 ± 0.1 °C
**S3** (February 2024)	250			8.7 ± 0.1 °C
**Sampled animals: 755**				
**Total subjects: 1775**				

**Table 2:** Viral foodborne pathogens, targeted genes, and primers used for nested RT-PCR assays.

Viral Pathogens	Genes	Reaction	Primers	Oligonucleotide Sequences (5′–3′)	Amplicon Size (bp)	References
**HAV**	VP1/2A junction	r	+2897	TATTCAGATTGCAAATTAYAAT	420 bp	[17]
			−3288	AAYTTCATYATTTTCATGCTCCT		
		n	+2949	TATTTGTCTGTYACAGAACAATCAG	244 bp	
			−3192	AGGRGGTGGAAGYACTTCATTTGA		
**HEV**	ORF-1	r	HEV-cs	TCGCGCATCACMTTYTTCCARAA	470 bp	[18]
			HEV-cas	GCCATGTTCCAGACDGTRTTCCA		
		n	HEV-csn	TGTGCTCTGTTGGCCCNTGGTTYG	333 bp	
			HEV-casn	CCAGGCTCACCRGARTGYTTCTTCCA		
	ORF-2	r	ORF2con-s1	GACAGAATTRATTTCGTCGGCTGG	197 bp	[19]
			ORF2con-a1	CTTGTTCRTGYTGGTTRTCATAATC		
		n	ORF2con-s2	GTYGTCTCRGCCAATGGCGAGC	145 bp	
			ORF2con-s2	GTTCRTGYTGGTTRTCATAATCCTG		
**NoV GI**	Capsid N/S domain	r	G1SKF	CTGCCCGAATTYGTAAATGA	330 bp	[20]
			G1SKR	CCAACCCARCCATTRTACA		
**NoV GII**			G2SKF	CNTGGGAGGGCGATCGCAA	387 bp	
			G2SKR	CCRCCNGCATRHCCRTTRTACAT		
**Norovirus GI-GII**	RdRp region	n	MR3	CCGTCAGAGTGGGTATGAA	470 bp	[21]
			MR4	AGTGGGTTTGAGGCCGTA		
		n	Yuri22F	ATGAATGAGGATGGACCCAT	370 bp	[22]
			Yuri22R	CATCATCCCCGTAGAAAGAT		

ORF: Overlapping open reading frames; r: reverse transcription; n: nested PCR; D = A, G, T; M = A or C; N = A, C, G, T; R = A or G; Y = C or T; bp: base pairs.

**Table 3:** Geographical distribution: viral RNA detections and prevalence values.

Farms	Viral Pathogens	Prevalence Values	CI 95%
**F1**	0/13 HAV	0.00%	-
	10/13 HEV	76.92%	54.02–99.82%
	3/13 NoV GI	23.07%	0.17–45.97%
**F2**	0/9 HAV	0.00%	-
	6/9 HEV	66.67%	35.88–97.46%
	3/9 NoV GI	33.33%	2.54–64.12%
**F3**	2/15 HAV	13.33%	1.13–25.53%
	3/15 HEV	20.00%	0.78–32.20%
	10/15 NoV GI	66.67%	42.82–90.52%

## Data Availability

The original contributions presented in the study are included in the article, further inquiries can be directed to the corresponding author.
